# Take Care

**Published:** 1871-03

**Authors:** 


### TAKE CARE.

The Dr. Hall mentioned in a February advertising page is no relation of ours. His carbolic preparations may have all the virtues they claim and many more to be hereafter discovered; but the editor has no experience in connection with their use. The advertisement was not seen until it was printed, and the advertiser claims that it was by mistake of the printer associated with Hall's Journal of Health. We do not exactly know how he figures it out, but take his word for it that it was a mistake on purpose, and so we let it go; but take occasion to remark that any medicine on the face of the earth, or under the earth, or above it, having the name of "Hall" to it, has no connection with the editor. This is a free country, too free for its own good, and some persons seem to think they have a right, so claimed at least, to call any poison they please by anybody's name, and to swear that it will remedy anything and everything, from a sore toe to an empty pocket, which last, we are free to say, is the most hateful malady that ever afflicted a human being, for, like itch and small-pox, it keeps everybody at a respectful distance from you; for nobody likes to speak to anybody who is as poor as Job's cat. But, returning to our namesake's carbolic preparations, we are free to say that all the combinations of pure carbolic acid have a great variety of uses, especially for promoting cleanliness, for arresting destructive decay, whether about a man's house or his person. We do not advise their internal use, except by the direction of an educated physician and of respectable associations.

